# Evolutionary transitions in diet influence the exceptional diversification of a lizard adaptive radiation

**DOI:** 10.1186/s12862-022-02028-3

**Published:** 2022-06-07

**Authors:** Mauricio Ocampo, Daniel Pincheira-Donoso, Ferran Sayol, Rodrigo S. Rios

**Affiliations:** 1 Departamento de Biología, Doctorado en Ciencias Biológicas, Ecología de Zonas Áridas (EZA), Universidad de la Serena, Casilla 554, La Serena, Chile; 2 Red de Investigadores en Herpetología-Bolivia, Los Pinos Zona Sur, Av. José Aguirre 260, La Paz, Bolivia; 3 Unidad de Zoología, Instituto de Ecología, Universidad Mayor de San Andrés, Casilla 10077-Correo Central, La Paz, Bolivia; 4 MacroBiodiversity Lab, School of Biological Sciences, Queen’s University Belfast, 19 Chlorine Gardens, Belfast, BT9 5DL UK; 5 Centre for Biodiversity and Environment Research, Department of Genetics, Evolution and Environment, University College London, London, UK; 6 Instituto de Investigación Multidisciplinario en Ciencia y Tecnología, Universidad de La Serena, La Serena, Chile

**Keywords:** Comparative phylogeny, Dietary evolution, Net diversification rate, Specialist, Generalist, Macroevolutionary sink, Liolaemidae

## Abstract

**Background:**

Diet is a key component of a species ecological niche and plays critical roles in guiding the trajectories of evolutionary change. Previous studies suggest that dietary evolution can influence the rates and patterns of species diversification, with omnivorous (animal and plant, ‘generalist’) diets slowing down diversification compared to more restricted (‘specialist’) herbivorous and carnivorous diets. This hypothesis, here termed the “dietary macroevolutionary sink” hypothesis (DMS), predicts that transitions to omnivorous diets occur at higher rates than into any specialist diet, and omnivores are expected to have the lowest diversification rates, causing an evolutionary sink into a single type of diet. However, evidence for the DMS hypothesis remains conflicting. Here, we present the first test of the DMS hypothesis in a lineage of ectothermic tetrapods—the prolific Liolaemidae lizard radiation from South America.

**Results:**

Ancestral reconstructions suggest that the stem ancestor was probably insectivorous. The best supported trait model is a diet-dependent speciation rate, with independent extinction rates. Herbivory has the highest net diversification rate, omnivory ranks second, and insectivory has the lowest. The extinction rate is the same for all three diet types and is much lower than the speciation rates. The highest transition rate was from omnivory to insectivory, and the lowest transition rates were between insectivory and herbivory.

**Conclusions:**

Our findings challenge the core prediction of the DMS hypothesis that generalist diets represent an ‘evolutionary sink’. Interestingly, liolaemid lizards have rapidly and successfully proliferated across some of the world’s coldest climates (at high elevations and latitudes), where species have evolved mixed arthropod-plant (omnivore) or predominantly herbivore diets. This longstanding observation is consistent with the higher net diversification rates found in both herbivory and omnivory. Collectively, just like the evolution of viviparity has been regarded as a ‘key adaptation’ during the liolaemid radiation across cold climates, our findings suggest that transitions from insectivory to herbivory (bridged by omnivory) are likely to have played a role as an additional key adaptation underlying the exceptional diversification of these reptiles across extreme climates.

**Supplementary Information:**

The online version contains supplementary material available at 10.1186/s12862-022-02028-3.

## Background

The evolutionary radiation of animal lineages can often be influenced by the adaptive diversification of their diets [1–5]. In fact, lineage proliferations triggered by ecological opportunity—vacant niche space—are largely determined by the advent of a novel resource, or a wide array of them, that promotes niche expansions that lead to intraspecific adaptive diversification [6–8] and ecological speciation [1, 9–11]. As these processes of diversification unfold, the emergence of new species can be facilitated by partition of niche space via transitions from generalist to specialist diets. Along this axis of dietary adaptive transitions, a range of biological components of species are also affected, including their spatial distribution, their position within trophic networks, the nature of life history trade-offs that balance energetic budgets, and their chances of persistence under rapidly changing environments [5, 9, 12–15]. Collectively, therefore, the interactions among these ecological phenomena are expected to influence processes of speciation and extinction, and thus, of diversification rates within lineages [5, 16].

A growing interest in establishing the role of dietary evolution in lineage diversification has led to the emergence of the intriguing hypothesis that omnivory (the consumption of both animals and plants) can drag species to an evolutionary sink [17, 18]. This ‘dietary macroevolutionary sink’ (DMS) hypothesis suggests that dietary specialists have an ecological advantage over omnivorous species given that the former are adapted to efficiently exploit a narrow set of resources, whereas the latter perform less efficiently across a wide range of different resources (a ‘jack of all trades is a master of none’ mechanism [17]). Consistent with this prediction, analyses performed in endotherms reveal that species with a more specialized diet, such as herbivory or insectivory, undergo significantly higher diversification rates than omnivores, in which extinction rates are higher and speciation rates lower [17, 18]. However, evolutionary transitions from a specialist to a generalist diet occur at much higher rates than in other directions [17, 18]. Therefore, this hypothesis posits that omnivory is a ‘macroevolutionary sink’ [17, 18]. Yet, evidence for the DMS hypothesis remains contested. For example, a study conducted on fish lineages revealed that omnivory is associated with the highest net diversification and transition rates [19]. In addition, the hypothesis’ core predictions oppose the classical early theory that diversification tends to transition from generalist ancestors to specialist descendants, leading niche specialists towards an evolutionary "dead end" as a result of major constrains involved in the return to an omnivorous diet [20–22].

Despite the critical role that dietary evolution plays in our understanding of the proliferation of biodiversity, the lack of studies across a wider range of lineages prevents a robust assessment of the generality of its predicted evolutionary outcomes. In fact, although reptiles (avian and non-avian) represent the most species-diverse lineage among modern tetrapods [23, 24], and their diets have been shown to be linked to processes of evolutionary radiation across contrasting environments [25–27], the DMS hypothesis remains untested among ectothermic tetrapods. Most studies on the diet of ectothermic tetrapods are limited to describing lists of items consumed by species [28–32]. Only a few studies have addressed the evolution and conservatism of trophic strategies at phylogenetic scales [25, 33, 34].

Reptiles offer ideal models to address hypotheses about the macroevolutionary links between trophic transitions and lineage diversification. These vertebrates span the whole range of the dietary spectrum, with a dominant tendency for animal consumption relative to a much lower frequency of herbivory [35]. Only a few families of lizards have strictly herbivorous species, most of which are large bodied and restricted to tropical regions [27, 36]. A remarkable exception to this ‘rule of reptilian herbivory’ is the South American lizard family Liolaemidae [25]. These lizards have rapidly diversified across a range of climates that mirror the climatic range occupied by all living lizards combined [37, 38]. As a result, liolaemids have evolved a wide range of dietary adaptations from strictly herbivores and carnivore specialists, to broadly generalists that even include cannibalism [39, 40]. Espinoza et al. [25] analyzed the recurrence and faster rate of herbivory in liolaemid lizards, revealing that these species break the ecophysiological rules of reptilian herbivory because they are small bodied and live in cool climates. Another feature of liolaemids is the variability of its three genera, with *Liolaemus* standing as one of nature’s most prolific adaptive radiations [37, 41, 42], to the genus *Ctenoblepharys* represented by a single species [43]. A recent study by Olave et al. [44], showed vast differences in diversification rates within the family’s genera, but the underlying factors of these differences remain elusive. One possibility is that some traits allow the use of resources more efficiently, triggering species diversification [45]. For instance, dietary niches might be a key factor driving species diversification [17–19], helping to explain the extreme variation in both diversification rates and dietary niches in the liolaemid family, especially if the effect of other possible traits such as habitat or parity mode is ruled out [46, 47].

In this study, we analyze a large-scale dataset spanning 185 species of the Liolaemidae family distributed across a wide range of ecological environments and climates, to address the DMS hypothesis of macroevolutionary diversification mediated by dietary transitions, and to assess the role that diet evolution has played in the diversification of this prolific lizard radiation. Therefore, if the DMS hypothesis holds in reptiles, we expect to observe a higher rate of net diversification in specialized (herbivory and insectivory) than generalist (omnivory) diets, and we predict that evolutionary transitions from a specialized to an omnivorous diet will occur at much higher rates than in other directions.

## Results

### Ancestral diet state reconstruction

**Fig. 1:**
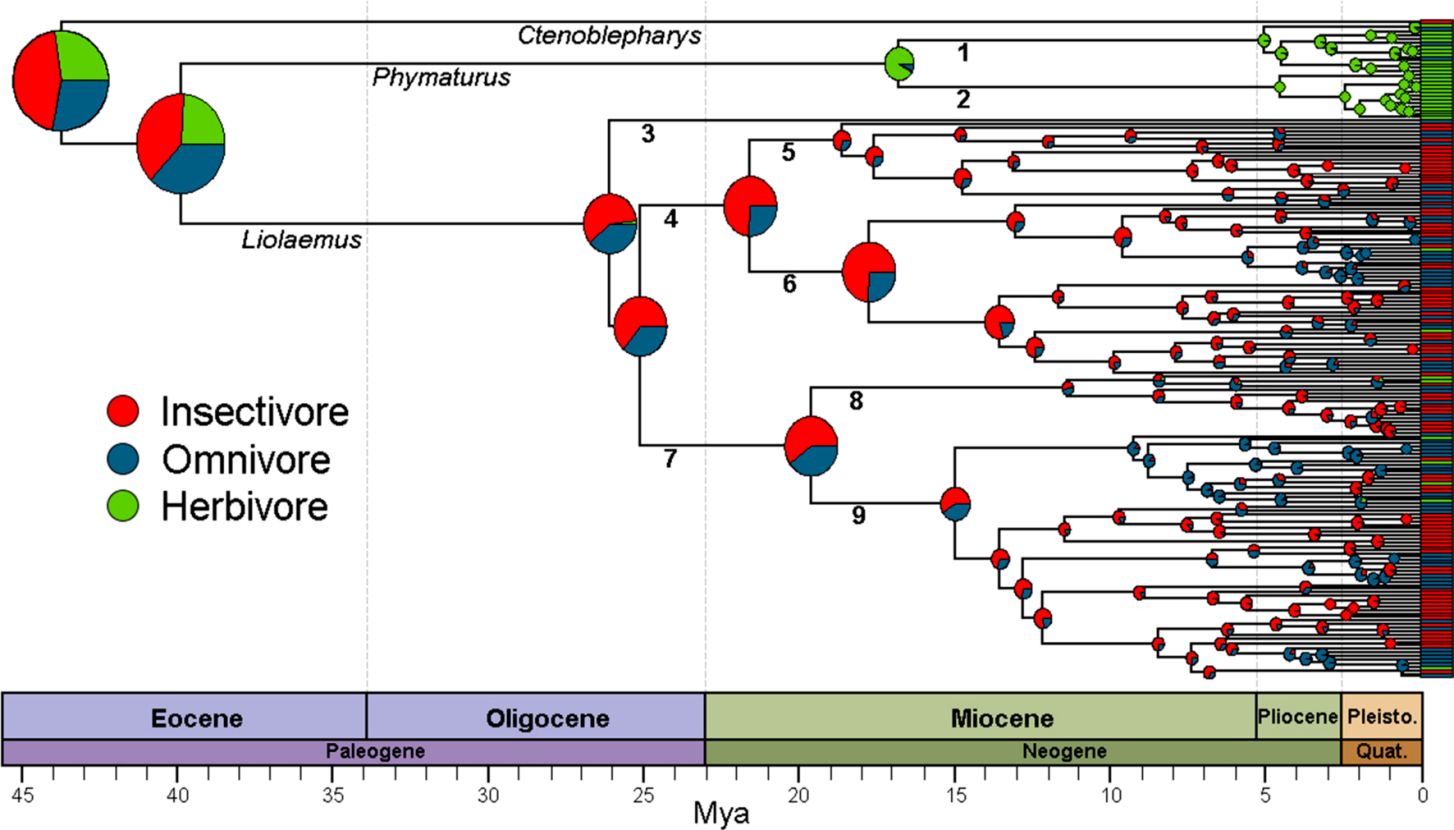
Ancestral reconstruction of dietary diversification throughout the Liolaemidae evolutionary history (pie charts at nodes represent posterior probabilities of each diet class), averaged across 100 trees. (1) *Phymaturus* *palluma* group; (2) *Phymaturus* *patagonicus* group; (3) *Liolaemus* *walkeri* group; (4) *Liolaemus* subgenus; (5) *Liolaemus* *nigromaculatus* section; (6) *Liolaemus* *chiliensis* section; (7) *Eulaemus* subgenus; (8) *Liolaemus* *lineomaculatus* series; (9) *Liolaemus* *montanus* series.

Based on trait state reconstruction (Fig. 1) with the Stochastic Character Mapping (SCM) method, the stem ancestor of the Liolaemidae family was more likely an insectivore (highest probability, p = 0.45, compared to an herbivore with p = 0.27, or an omnivore with p = 0.28). The common ancestor of *Phymaturus* + *Liolaemus*, show a high probability to have been an omnivore or insectivore (p = 0.37 and p = 0.39, respectively). Herbivory originated ~ 17 to 39 million years ago, and has remained predominantly invariable within *Phymaturus*. Among *Liolaemus* species, insectivory predominated in the earliest ancestors, herbivory has converged on nine occasions within the clade, and in all of them the insectivorous ancestor went through an omnivorous transition. Clades with both herbivory and omnivory diets increased their diversification from the Pliocene (a little more than 5 Mya) onwards.

### Effect of diet type on diversification dynamics

The diversification analysis using Several Examined and Concealed States-dependent Speciation and Extinction (SecSSE) shows that the best supported model is a diet-dependent speciation rate, with independent extinction rates, no hidden states and varying transitions rates among diet types (Table 1). Under this model, herbivores have almost three times higher speciation rates (λ = 0.453) compared to insectivores (λ = 0.167), whereas omnivores have an intermediate rate (λ = 0.285). Extinction is invariable across the phylogeny, with a value of μ = 0.014. In all cases, models not including hidden states were better supported, suggesting that the effect of diet on diversification is not spurious (Table 1). Similarly, models allowing variation of transition rates among character states had better support than models assuming equal rates between character states (Table 1).

**Table 1:** Model comparison for independent and dependent diet diversification, including models with hidden traits or with equal rates of transition

Model type	Hidden trait (A/B)	Equal transition rates (Q)	Speciation rates (λ_i_)	Extinction rates (μ_i_)	Transition rates (Q_i_)	Sum of params	LogLik	AICc
Diet independent speciation and extinction rates	No	Yes	1	1	1	3	− 675.3	1356.7
	No	No	1	1	6	8	− 659.5	1335.9
	Yes	Yes	1	1	1	3	− 701.2	1408.6
	Yes	No	1	1	18	20	− 701.2	1447.6
Diet-dependent speciation and independent extinction rate	No	Yes	3	1	1	5	− 675.1	1360.5
	**No**	**No**	**3**	**1**	**6**	**10**	− **655.5**	**1332.2**
	Yes	Yes	6	1	1	8	− 857.8	1732.4
	Yes	No	6	1	18	25	− 788.0	1634.2
Independent speciation and diet-dependent extinction rates	No	Yes	1	3	1	5	− 670.5	1351.2
	No	No	1	3	6	10	− 656.8	1335.0
	Yes	Yes	1	6	1	8	− 794.2	1598.7
	Yes	No	1	6	18	25	− 795.2	1648.5
Diet-dependent speciation and extinction rates	No	Yes	3	3	1	7	− 663.9	1342.5
	No	No	3	3	6	12	− 659.5	1344.8
	Yes	Yes	6	6	1	13	− 839.1	1706.4
	Yes	No	6	6	18	30	− 805.6	1683.4

Values in bold indicate the best model

Number of inferred rates for speciation (λ), extinction (μ) and transition (Q) are specified in each case, and LogLik and AICc values are shown

### Diet-dependent speciation using MuSSE

**Fig. 2:**
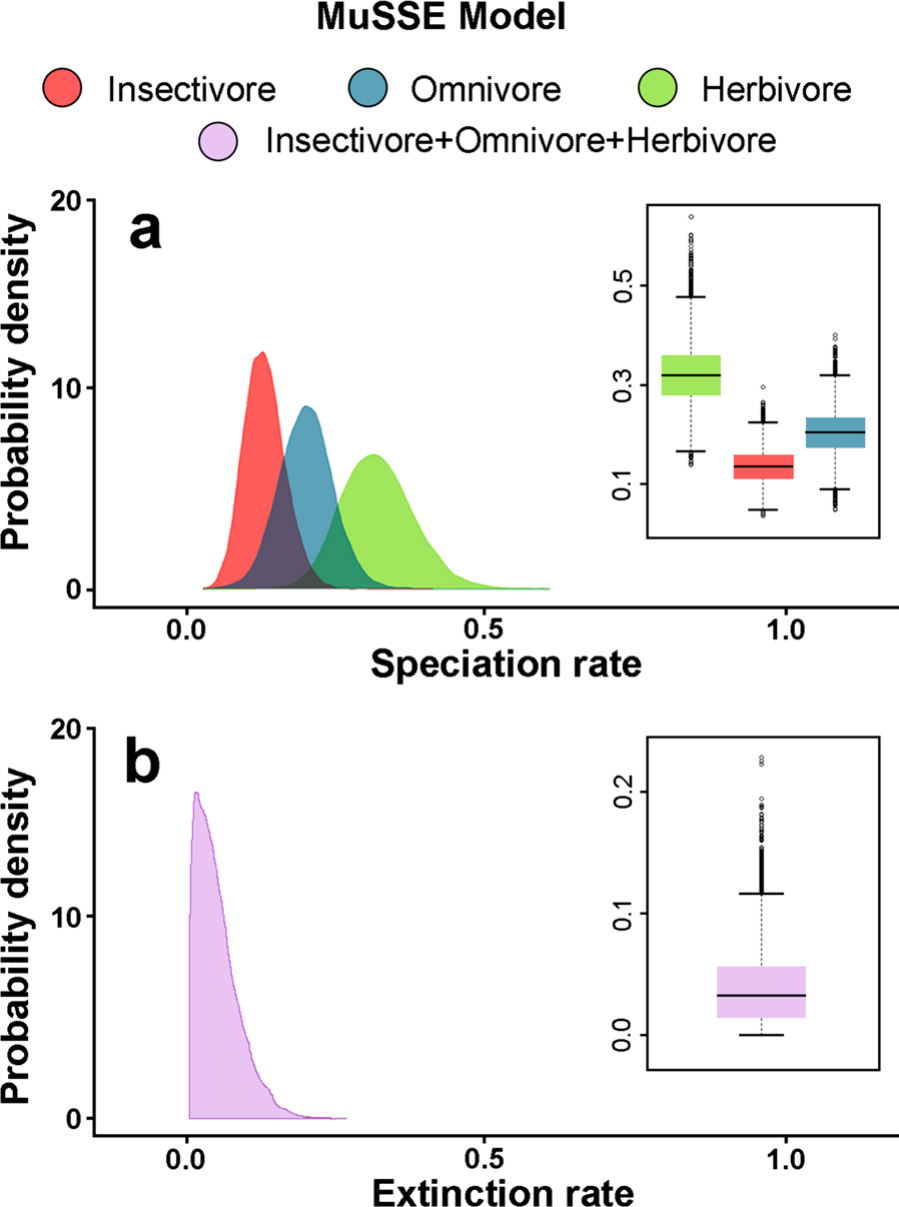
Distribution patterns of evolutionary rates across diets of the liolaemid family. Probability densities from the MuSSE model for **a** speciation rates associated with different dietary types, herbivory (green), omnivory (blue), and insectivory (red), and for **b** extinction rates across all the trees. Box plots within panels show the variation of rates in quartiles within and across diets. Different diet types show significant differences as compared with EMMs adjusted by means of the Tukey as a post-hoc test

To better account for uncertainty in parameter estimates, we repeated the best model suggested by SecSSE using a Multistate Speciation and Extinction Model (MuSSE) analysis. This model confirms previous findings that herbivores had the highest speciation rates, followed by omnivores, and insectivores had the lowest rate (Fig. 2a). As the extinction rate is the same for all three diet types (Fig. 2b), net diversification rates follow the same patterns as speciation rates, from highest in herbivores to lowest in insectivores. Differences in both speciation and diversification rates are statistically significant in both cases. Speciation Likelihood Ratio Test (LRT) (*X*^*2*^ = 175.21, p < 0.0001), Net diversification LRT (*X*^*2*^ = 452.63, p < 0.0001).

### Transition rates among diet types

**Fig. 3:**
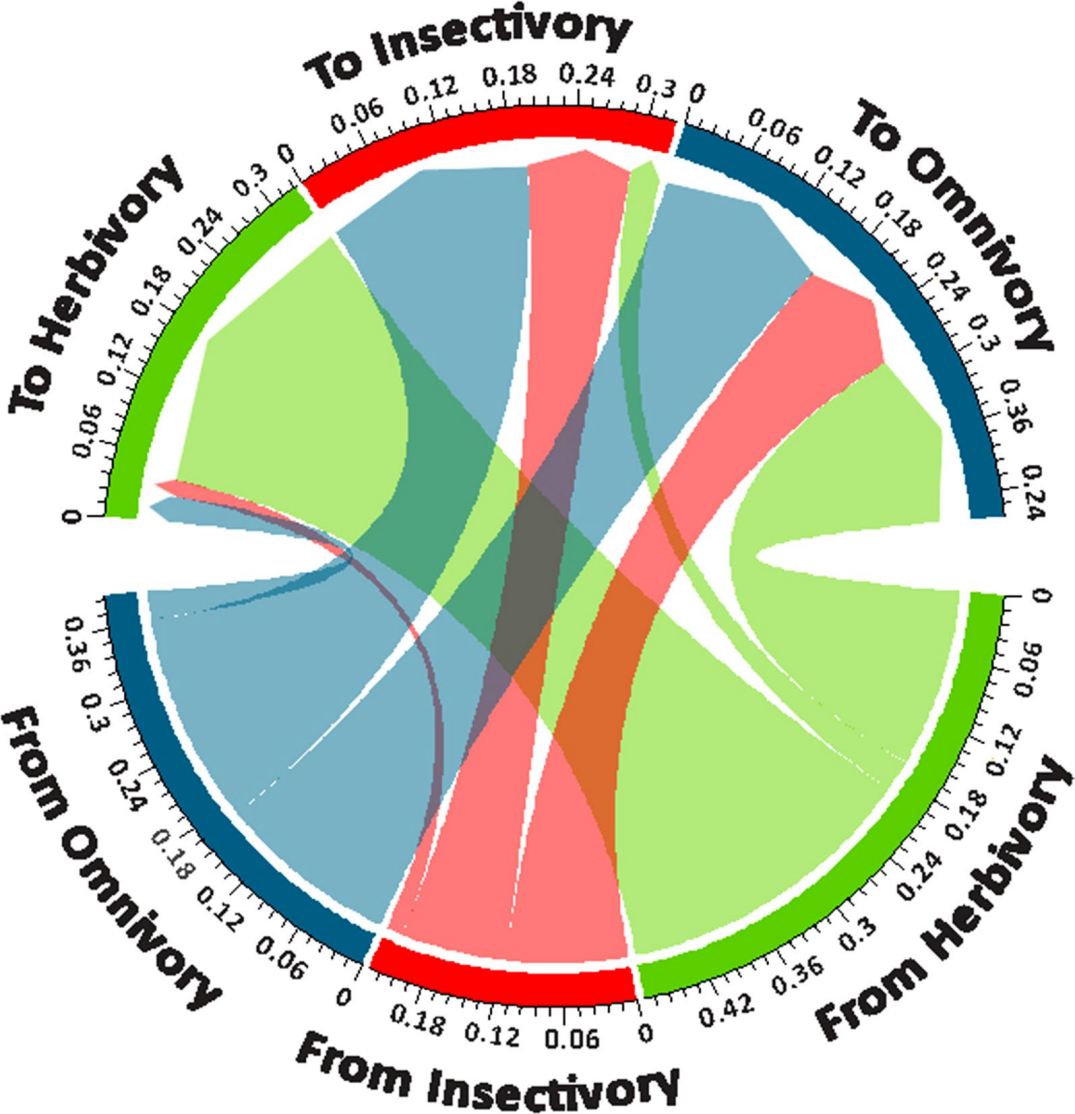
Trait conservatism and transition rates estimated across dietary states, where values are total cumulative rates for each diet type. The lower half of the circle shows the proportion that each diet contributes to trait conservatism (within the same diet) and transition to other diet types, and the upper half of the circle shows the contribution that each diet receives from transition events, whether is from its own dietary state (a trait conservatism process) or from other diets during all the speciation events on the phylogenetic tree

In the MuSSE model, herbivory has the highest trait conservatism and transition rate estimates to other diets (0.52—i.e., the mean value of the posterior distribution of the corresponding rate) (lower half, Fig. 3). From this diet, almost 31% of transitions (0.16) go into omnivory, and only 6% (0.03) to insectivory. The remaining 61% (0.32) are conserved, that is, lineages maintained as herbivores. In omnivory, trait conservatism and transition rates estimated to other diets is 0.43, where 46% (0.20) retain this form of diet, 46% (0.20) transition to insectivory, representing the highest rate of transition between diets; and only 7% (0.03) transition to herbivory. Insectivory has the lowest trait conservatism and transition rate (0.27) to other diets, where 51% (0.11) transition to omnivory, 39% (0.14) remain as insectivory, and only 7% (0.02) change to herbivory; this transition is the lowest among diet types. All rates in trait conservatism and dietary transitions show statistically significant differences based on the EMMs adjusted by Tukey method and the LRT from the GLM analysis (*X*^*2*^ = 305.5, p < 0.0001).

In general, more specialized diets (i.e., insectivory and herbivory) have switched more often into omnivory or remain in their own state across time than they have switched between each other (upper half, Fig. 3). Herbivory is more supported by trait conservation than by transitions with other diets. Omnivory, however, contributes more to insectivory by transition than the conservation of the insectivorous trait itself, this process has allowed the insectivorous diet to exist throughout the liolaemid evolutionary history.

## Discussion

Our study provides the first test of the DMS hypothesis—that evolutionary transitions in diet influence diversification of animal lineages—performed in ectothermic tetrapods. Using the prolific Liolaemidae lizard radiation, our evidence challenges the hypothesis’s core prediction that omnivory acts as a macroevolutionary sink. In contrast with this prediction, we observed that omnivory ranks second in net diversification rate and contributes to insectivory through the highest evolutionary transitions. Although the net diversification rate in insectivores is the lowest among diet types, insectivory is present in more species than herbivory or omnivory (84, 33, 68 species, respectively). These findings challenge the preconception that traits with high net diversification rates have high species diversity [44, 48].

Our results suggest that insectivory is likely to have been promoted by omnivory, and favored by a low extinction rate. Similar patterns have been observed in birds [17] where transitions from omnivory to insectivory occur at higher rates than its own speciation and net diversification rates, resulting in the most species-rich diet. Therefore, omnivory plays an important role in the transition across specialists by acting as an evolutionary stepping-stone, rather than as a macroevolutionary sink, as predicted by the DMS hypothesis [17, 18]. Similarly, a study on fish suggested that trophic versatility can sustain high diversification rates, relax interspecific competition, and facilitate the local co-occurrence of ecologically similar species [19]. We observed this versatility to be the case in *Liolaemus* lizards—the second most species-rich genus of lizards in the Neotropics, and which spans all types of diets. In fact, the rapid adaptive radiation undergone by *Liolaemus* across a remarkable range of environments throughout South America has been suggested to be associated with their exceptional adaptive versatility in multiple components of their phenotypes [8, 37, 38, 42, 44, 49–52].

Our observation that herbivory is associated with the highest rates of speciation, aligns with the patterns of speciation rate observed by Olave et al. [44] in *Phymaturus*. However, in this study, high extinction rates in *Phymaturus* rank this group second in net diversification rate [44]. This may be related to the fact that this genus is dominated by strong niche conservatism with species sharing multiple key ecological and life history traits, including their adaptation to cold climates, their occupation of rocky outcrops, their herbivorous diets, viviparous reproductions, and extreme low fecundity [26, 46, 49, 53, 54]. Conversely, herbivory in *Liolaemus* unlike *Phymaturus* evolved convergently (nine times), and in all cases the insectivore ancestor had to undergo an omnivorous transition; thus, it is likely that the common ancestor of both *Phymaturus* and *Liolaemus* was probably an omnivore.

### Herbivory in lizards

Herbivory in lizards is uncommon relative to other diet types, found in only 5% of species [55], A key explanation for such rarity is that plant-consumption provides poorly nutritious, and difficult to digest food resource [56]. For example, herbivores must be highly efficient to maintain a constant high body temperature, and form symbiotic associations with bacteria, fungi, and protozoa [25, 57–59]. Remarkably, Liolaemidae contains the highest prevalence of herbivory among living reptiles [25, 35] (33 species in this study), a dietary specialization that our results show to have the highest speciation rates. These findings may be linked to the direct effects that The Andes are believed to have exerted on the evolutionary radiation of liolaemid lizards, triggered by the vast-scale emergence of ecological opportunity that the active uplifts of the mountains provided over the past ~ 20–30 million years [26, 37, 38, 42]. Given that another feature that comes with Andean organisms is their predominantly small ranges [60], as it is the case with liolaemid lizards [26, 37, 42, 61], their evolutionary history tightly linked to the rapidly changing topography (and climate) of these mountains is likely to have led to active episodes of extinctions at the same time.

The patterns in transition rates we found between diets are contrary to the hypothesis that specialization is a “dead end” [19, 62–64]. Moreover, transition rates from specialists to omnivores are similar to the net diversification rates of specialists. In fact, the transition rate from insectivory to omnivory is greater than the net diversification rate of insectivory, which suggests that specialized diets may have a high probability of transitioning to another diet type also. Herbivory contributes more species to omnivory than insectivory, a result that was also found in mammals [18] and birds [17], and the transition rate from omnivory to insectivory is higher than to herbivory, which is similar in birds [17]. Perhaps because it is physiologically easier for an herbivore to process protein again, than for an insectivore to change the digestive tract to receive and process plant material, which is difficult to digest because of the low levels of essential nutrients [65, 66]. Plants often defend themselves with toxins, and associations with microbes or symbiotic nematodes are necessary to improve digestion [57, 58]. Diet specialization depends on the degree of interactions between intrinsic traits of individuals and ecological contexts [67], and some clades with highly specialized diets will hardly transition to another type of diet. However, with our results we show that there is a certain degree of specialization that still has the possibility of transitioning to completely opposite diets through an intermediate step.

### Paleoecological history

During the mid-Eocene, southern South America recorded a more seasonal climate and open areas dominated by grasslands appeared, probably associated with a moisture gradient [68–70]. This environmental change has been proposed to be linked to a shift in species dietary composition in mammals, where insectivores switched to an herbivorous diet [69, 71]. Similarly, a detailed analysis of the content in a Eocene bird fossil revealed the presence of plant remains, concluding that this species had a facultative herbivorous diet [72]. The genus *Phymaturus*, which consists of almost exclusively herbivores, diverged in the late Eocene ~ 40 Mya, and it is estimated to have originated in Patagonia, and central Andes [37], an area with a habitat type that is consistent with the presence of open areas dominated by grasslands in the mid Eocene [70, 73]. For this reason, like mammals and birds, it is possible that the appearance of open areas dominated by grasslands in the Eocene triggered the transition to an herbivorous diet in Liolaemidae.

## Conclusions

Our findings challenge the core prediction of the DMS hypothesis that generalist diets represent an "evolutionary sink". Unlike other groups, omnivory in liolaemids plays an important role in diversification and transition from other diets (i.e., insectivory and herbivory). Potentially as a consequence of this versatility, this family has rapidly and successfully proliferated in a variety of climates throughout its altitudinal and latitudinal range in southern South America. Therefore, just like the evolution of viviparity in liolaemids has been regarded as a "key adaptation" underlying the prolific radiation of these reptiles across cold climates, our findings suggest that transitions through omnivory are likely to have played an important role as an additional key adaptation that has facilitated their rapid evolution across such extreme environments.

## Methods

### Taxon sampling and phylogenetic tree

To perform phylogenetic analyses, we used the calibrated tree in Esquerré et al. [37], based on six nuclear (*B1D*, *EXPH5*, *KIF24*, *MXRA5*, *PLRL*, *PNN*) and four mitochondrial loci (*cytb*, *12S*, *ND2*, *ND4*) as molecular markers. Their tree is based on a GTR + G for the best gene partitioning scheme and substitution model. Fossils representing the earliest record of the *Eulaemus* clade in the Early Miocene were used to place a mean prior on the tree height of this subgenus. The tree covers almost 70% (1 *Ctenoblepharys*, 188 *Liolaemus*, 35 *Phymaturus*) of the current species of Liolaemidae [23]. For more details on the time-calibrated phylogenetic tree see Esquerré et al. [37].

### Diet data compilation

We compiled dietary data for 185 liolaemid species (33 herbivores, 84 insectivores, and 68 omnivores). This accounts for 55% of representativeness of the family (Additional file 1: Table S1). We based our diet data compilation on Meiri [55], which contains an extensive revision of lizard life history traits, and many of these publications include the type of diet without any other details. One exception is Espinoza et al. [25], who made one of the first classifications of diet types based on proportion of consumed items. We found this to be very helpful, however their classification of the omnivore (11–50% volumetric proportion of plant matter in the diet) and herbivore groups (70–100%) left an unclassified gap between 50 and 70% of plant matter, making the boundary between these two diet groups uncertain [37]. Subsequent studies adopted this classification adjusting omnivory to 11–50% and herbivory to > 50% [55, 74], but we believe that in this arrangement, omnivory is underestimated and herbivory is overestimated. Therefore, we considered an insectivorous species when up to 10% plant matter was found in the stomach content, omnivory is better represented between 11 and 75% of plant matter, and that a fundamental plant diet (herbivorous) is > 75% [75]. Using these criteria, a more detailed literature revision, and personal data (i.e., records of stomach contents) gathered by one of us (DPD), we found 8 species that needed to be changed from herbivore to omnivore (see details in Additional file 1: Table S1).

### Phylogenetic comparative methods

#### Ancestral diet state reconstruction

To reconstruct ancestral diet states and evaluate their historical shifts across the evolution of Liolaemidae, we implemented Stochastic Character Mapping (SCM) [76] on the Maximum Clade Credibility (MCC) Phylogenetic Tree using the *make.simmap* function from the *phytools* package [77] within the statistical environment R [78]. SCM is a Bayesian approach that generates a posterior probability distribution, based on Maximum Likelihood (ML), of the ancestral states of diet and their transition times across the branches of the MCC tree by way of Markov Chain Monte Carlo (MCMC) [79]. Before running the SCM, we selected the best evolutionary model for character distribution across the tree by comparing three different models: (1) an equal-rates model “ER”, where a single parameter governs all transition rates, (2) a symmetric model “SYM”, where forward and reverse transitions share the same parameter, and (3) an all-rates-are different model “ARD”, where each rate is a unique parameter. Models were ran using the *FitMk* function from *phytools* [77]. Finally, we selected “SYM” as the best model according to the Akaike Information Criterion (AIC). All models were built using 100 simulated trees.

### Effect of diet type on diversification dynamics

To test the influence of diet on species diversification dynamics, we compared different models of diversification using Several Examined and Concealed States-dependent Speciation and Extinction, using the *SecSSE* R package [47]. This method allows to fit different character-dependent speciation and extinction, where rates may vary across different character states. In addition, one can also include “hidden” traits that could influence diversification independently of the trait of interest. The values of each speciation (λ) and extinction (μ) rates are estimated simultaneously with transition rates among character states (Q) by maximum likelihood approach. In our case, we tested the hypothesis that different diets (herbivore, insectivore, omnivore) have different rates of speciation and extinction. To do so, we fitted four types of models: (i) λ and μ are independent of the diet type, (ii) λ depends on diet while μ is independent, (iii) λ is independent while μ depends on the diet type and (iv) both λ and μ can vary with diet type. These four combinations where then repeated with and without constraining Q to be equal between character states or allowing rates to vary. In addition, all models were repeated while including a hidden trait with two states (A/B), to assess whether associations between diet and diversification might be spurious. Therefore, we compared a total of 16 different models combining diet-dependent and independent λ and μ, equal or different Q and including or not hidden states. When concealed (i.e., hidden) states are included, dual transitions are set to zero, so we do not allow transitions from diet and hidden states happening simultaneously. SecSSE models also allow to account for differences in species sampling among character states. In our case, our study includes 185 species with known phylogenetic relationships and diet out of 338 species in the clade [23], with a sampling proportion (F) for each diet as F_herbivore_ = 0.46, F_insectivore_ = 0.58 and F_omnivore_ = 0.57. The models were set to run for 50,000 iterations and all models reached convergence. The fit of each model was compared using the AICc criteria.

### Diet-dependent speciation using MuSSE

In order to better account for parameter uncertainty in the speciation and extinction rates, we repeated the best model from the SecSSE analysis using a Bayesian approach, implemented in the Multi-State Speciation and Extinction (MuSSE) models from the *diversitree* R package [78, 80]. We ran a model where speciation rates may vary among character states (i.e., diet types), maintaining a single extinction rate among states. The character is modeled as evolving under a constant rate Markov model of evolution (mcmc function). Models were ran using 10,000 iterations through a Markov Chain Monte Carlo (MCMC) process, using an exponential prior and a tuning parameter for the sampler (w) that was pre-calibrated with 100 Bayesian parameter estimation MCMCs. To test for differences in speciation, extinction, diversification, and transition rates among diet strategies, we used independent GLMs with a gaussian distribution and identity link function. Parameter estimates of the models were evaluated for statistical significance based on LRT. Models considered the estimated rate as the response variable and diet with three levels (insectivore, herbivore and omnivore) as the independent variable. Finally, we conducted EMMs adjusted by means of the Tukey method [81] as post-hoc comparison using the *emmeans* package [82].

## Supplementary Information

**Additional file 1: Table S1. **Species, diet, and bibliography consulted.

## Data Availability

All data generated or analyzed during this study are included in this published article [and its Additional information files].

## Abbreviations

SecSSE: Several examined and concealed states-dependent speciation and extinction
MCC: Maximum clade credibility
DMS: Dietary macroevolutionary sink
Mya: Million years ago
LRT: Likelihood ratio test
MuSSE: Multistate Speciation and Extinction Model
MCMC: Markov Chain Monte Carlo
SCM: Stochastic character mapping
ML: Maximum likelihood
ER: Equal-rates model
SYM: Symmetric model
ARD: All-rates-are different model
AIC: Akaike information criterion
GLM: General linear models
EMMs: Estimated marginal means
