# Supplementary material for: Evolutionary transitions in diet influence the exceptional diversification of a lizard adaptive radiation
Source: BMC Ecol Evol. 2022 Jun 7;22:74. doi: 10.1186/s12862-022-02028-3 (PMC9175459; doi:10.1186/s12862-022-02028-3)
Supplement: Supplementary file 1 — Additional file 1: Table S1. Species, diet, and bibliography consulted. [file 12862_2022_2028_MOESM1_ESM.docx]

**Additional file 1**

**Table S1 Species, diet, and bibliography consulted**

| **Species** | **Diet** | **Resource** |
| --- | --- | --- |
| *Ctenoblepharys adspersa* | Insectivorous | [1] |
| *Liolaemus abaucan* | Omnivorous | [1] |
| *Liolaemus albiceps* | Herbivorous | [1-4] |
| *Liolaemus alticolor* | Omnivorous | [1]* |
| *Liolaemus andinus* | Omnivorous | [1] |
| *Liolaemus annectens* | Omnivorous | [1] |
| *Liolaemus anomalus* | Insectivorous | [1] |
| *Liolaemus araucaniensis* | Omnivorous | [1] |
| *Liolaemus archeforus* | Omnivorous | [1] |
| *Liolaemus atacamensis* | Insectivorous | [1]* |
| *Liolaemus audituvelatus* | Insectivorous | [1] |
| *Liolaemus austromendocinus* | Insectivorous | [1] |
| *Liolaemus azarai* | Insectivorous | [1] |
| *Liolaemus baguali* | Insectivorous | [1] |
| *Liolaemus bellii* | Omnivorous | [1]* |
| *Liolaemus bibronii* | Insectivorous | [1] |
| *Liolaemus bitaeniatus* | Insectivorous | [1] |
| *Liolaemus boulengeri* | Insectivorous | [1] |
| *Liolaemus buergeri* | Omnivorous | [1, 2, 5-9] + |
| *Liolaemus burmeisteri* | Insectivorous | [1] |
| *Liolaemus calchaqui* | Omnivorous | [1] |
| *Liolaemus canqueli* | Omnivorous | [1] |
| *Liolaemus capillitas* | Insectivorous | [1] |
| *Liolaemus chacabucoense* | Insectivorous | [1] |
| *Liolaemus chacoensis* | Insectivorous | [1] |
| *Liolaemus chaltin* | Omnivorous | [1] |
| *Liolaemus chehuachekenk* | Omnivorous | [1] |
| *Liolaemus chiliensis* | Insectivorous | [1]* |
| *Liolaemus chillanensis* | Omnivorous | [1] |
| *Liolaemus coeruleus* | Insectivorous | [1] |
| *Liolaemus confusus* | Insectivorous | [1] |
| *Liolaemus constanzae* | Omnivorous | [1]* |
| *Liolaemus crandalli* | Omnivorous | [1] |
| *Liolaemus crepuscularis* | Omnivorous | [1, 10] + |
| *Liolaemus cristiani* | Omnivorous | [1] |
| *Liolaemus curicensis* | Insectivorous | [1]* |
| *Liolaemus curis* | Insectivorous | [1] |
| *Liolaemus cuyanus* | Omnivorous | [1] |
| *Liolaemus cuyumhue* | Insectivorous | [1] |
| *Liolaemus cyanogaster* | Insectivorous | [1] |
| *Liolaemus darwinii* | Insectivorous | [1] |
| *Liolaemus dicktracyi* | Insectivorous | [1] |
| *Liolaemus donosobarrosi* | Insectivorous | [1] |
| *Liolaemus dorbignyi* | Omnivorous | [1] |
| *Liolaemus eleodori* | Omnivorous | [1] |
| *Liolaemus elongatus* | Omnivorous | [1] |
| *Liolaemus escarchadosi* | Insectivorous | [1] |
| *Liolaemus espinozai* | Insectivorous | [1] |
| *Liolaemus etheridgei* | Omnivorous | [1] |
| *Liolaemus fabiani* | Omnivorous | [1]* |
| *Liolaemus famatinae* | Omnivorous | [1] |
| *Liolaemus fitzgeraldi* | Insectivorous | [1] |
| *Liolaemus fitzingerii* | Omnivorous | [1] |
| *Liolaemus fuscus* | Insectivorous | [1] |
| *Liolaemus gallardoi* | Omnivorous | [1] |
| *Liolaemus goetschi* | Insectivorous | [1] |
| *Liolaemus gracilis* | Insectivorous | [1] |
| *Liolaemus gravenhorstii* | Insectivorous | [1] |
| *Liolaemus grosseorum* | Insectivorous | [1] |
| *Liolaemus gununakuna* | Insectivorous | [1] |
| *Liolaemus hatcheri* | Herbivorous | [1, 2, 11, 12] |
| *Liolaemus heliodermis* | Omnivorous | [1] |
| *Liolaemus hellmichi* | Insectivorous | [1]* |
| *Liolaemus hermannunezi* | Insectivorous | [1] |
| *Liolaemus huacahuasicus* | Herbivorous | [1, 2, 13] |
| *Liolaemus inacayali* | Insectivorous | [1] |
| *Liolaemus insolitus* | Insectivorous | [1] |
| *Liolaemus irregularis* | Omnivorous | [1] |
| *Liolaemus isabelae* | Insectivorous | [1] |
| *Liolaemus janequeoae* | Insectivorous | [1, 14] |
| *Liolaemus kingii* | Omnivorous | [1] |
| *Liolaemus kolengh* | Insectivorous | [1] |
| *Liolaemus koslowskyi* | Insectivorous | [1] |
| *Liolaemus kriegi* | Omnivorous | [1] |
| *Liolaemus laurenti* | Omnivorous | [1] |
| *Liolaemus lavillai* | Omnivorous | [1] |
| *Liolaemus leftrarui* | Insectivorous | [1] |
| *Liolaemus lemniscatus* | Insectivorous | [1]* |
| *Liolaemus leopardinus* | Omnivorous | [1]* |
| *Liolaemus lineomaculatus* | Omnivorous | [1, 2, 15, 16] + |
| *Liolaemus loboi* | Insectivorous | [1] |
| *Liolaemus lorenzmuelleri* | Insectivorous | [1] |
| *Liolaemus lutzae* | Omnivorous | [1, 2, 17-23] + |
| *Liolaemus magellanicus* | Omnivorous | [1] |
| *Liolaemus maldonadae* | Insectivorous | [1] |
| *Liolaemus mapuche* | Insectivorous | [1] |
| *Liolaemus martorii* | Insectivorous | [1] |
| *Liolaemus melaniceps* | Omnivorous | [1] |
| *Liolaemus melanops* | Omnivorous | [1] |
| *Liolaemus molinai* | Herbivorous | [1, 24, 25] |
| *Liolaemus monticola* | Insectivorous | [1]* |
| *Liolaemus morenoi* | Omnivorous | [1] |
| *Liolaemus multicolor* | Omnivorous | [1] |
| *Liolaemus multimaculatus* | Insectivorous | [1] |
| *Liolaemus neuquensis* | Omnivorous | [1] |
| *Liolaemus nigrocoeruleus* | Insectivorous | [1] |
| *Liolaemus nigromaculatus* | Omnivorous | [1]* |
| *Liolaemus nigroviridis* | Omnivorous | [1]* |
| *Liolaemus nitidus* | Omnivorous | [1, 2, 25-27]* + |
| *Liolaemus occipitalis* | Omnivorous | [1, 28] |
| *Liolaemus olongasta* | Omnivorous | [1] |
| *Liolaemus orientalis* | Herbivorous | [1, 2, 11] |
| *Liolaemus ornatus* | Omnivorous | [1] |
| *Liolaemus pagaburoi* | Omnivorous | [1] |
| *Liolaemus parvus* | Insectivorous | [1, 29] |
| *Liolaemus paulinae* | Insectivorous | [1]* |
| *Liolaemus petrophilus* | Insectivorous | [1] |
| *Liolaemus pictus* | Omnivorous | [1]* |
| *Liolaemus platei* | Insectivorous | [1] |
| *Liolaemus poconchilensis* | Insectivorous | [1] |
| *Liolaemus poecilochromus* | Herbivorous | [1, 11, 30] |
| *Liolaemus pseudoanomalus* | Insectivorous | [1] |
| *Liolaemus pseudolemniscatus* | Insectivorous | [1] |
| *Liolaemus puna* | Omnivorous | [1, 2, 25, 31] + |
| *Liolaemus punmahuida* | Omnivorous | [1] |
| *Liolaemus quilmes* | Insectivorous | [1] |
| *Liolaemus ramirezae* | Omnivorous | [1] |
| *Liolaemus riojanus* | Insectivorous | [1] |
| *Liolaemus robertmertensi* | Insectivorous | [1] |
| *Liolaemus rothi* | Insectivorous | [1] |
| *Liolaemus ruibali* | Omnivorous | [1] |
| *Liolaemus sagei* | Insectivorous | [1] |
| *Liolaemus salinicola* | Insectivorous | [1] |
| *Liolaemus sarmientoi* | Omnivorous | [1] |
| *Liolaemus saxatilis* | Insectivorous | [1] |
| *Liolaemus scapularis* | Insectivorous | [1] |
| *Liolaemus schroederi* | Insectivorous | [1]* |
| *Liolaemus scorialis* | Omnivorous | [1] |
| *Liolaemus senguer* | Insectivorous | [1] |
| *Liolaemus septentrionalis* | Insectivorous | [1] |
| *Liolaemus signifer* | Omnivorous | [1, 2, 10, 25, 32] + |
| *Liolaemus silvai* | Insectivorous | [1] |
| *Liolaemus silvanae* | Herbivorous | [1, 2] |
| *Liolaemus sitesi* | Omnivorous | [1] |
| *Liolaemus somuncurae* | Insectivorous | [1] |
| *Liolaemus stolzmanni* | Insectivorous | [1] |
| *Liolaemus tari* | Insectivorous | [1] |
| *Liolaemus tehuelche* | Insectivorous | [1] |
| *Liolaemus telsen* | Insectivorous | [1] |
| *Liolaemus tenuis* | Insectivorous | [1] |
| *Liolaemus torresi* | Insectivorous | [1] |
| *Liolaemus tregenzai* | Herbivorous | [1, 2, 33] |
| *Liolaemus tristis* | Insectivorous | [1] |
| *Liolaemus umbrifer* | Omnivorous | [1] |
| *Liolaemus uniformis* | Omnivorous | [1] |
| *Liolaemus uptoni* | Insectivorous | [1] |
| *Liolaemus uspallatensis* | Insectivorous | [1] |
| *Liolaemus valdesianus* | Omnivorous | [1]* |
| *Liolaemus vallecurensis* | Omnivorous | [1] |
| *Liolaemus velosoi* | Insectivorous | [1] |
| *Liolaemus villaricensis* | Herbivorous | [1, 6, 25] |
| *Liolaemus walkeri* | Omnivorous | [1] |
| *Liolaemus wiegmannii* | Insectivorous | [1] |
| *Liolaemus xanthoviridis* | Omnivorous | [1] |
| *Liolaemus yanalcu* | Insectivorous | [1] |
| *Liolaemus zabalai* | Omnivorous | [1, 25, 34] + |
| *Liolaemus zapallarensis* | Omnivorous | [1]* |
| *Liolaemus zullyae* | Insectivorous | [1] |
| *Phymaturus aguedae* | Herbivorous | [1, 35] |
| *Phymaturus antofagastensis* | Herbivorous | [1, 2, 11, 36, 37] |
| *Phymaturus bibronii* | Herbivorous | [1, 25, 38] |
| *Phymaturus calcogaster* | Herbivorous | [1, 2, 12, 15] |
| *Phymaturus ceii* | Herbivorous | [1, 2] |
| *Phymaturus delheyi* | Omnivorous | [1] |
| *Phymaturus denotatus* | Herbivorous | [1, 39] |
| *Phymaturus dorsimaculatus* | Herbivorous | [1, 3] |
| *Phymaturus extrilidus* | Herbivorous | [1, 40] |
| *Phymaturus indistinctus* | Omnivorous | [1] |
| *Phymaturus mallimaccii* | Herbivorous | [1, 2] |
| *Phymaturus manuelae* | Herbivorous | [1, 41] |
| *Phymaturus nevadoi* | Herbivorous | [1, 7] |
| *Phymaturus palluma* | Herbivorous | [1, 20, 42-45] |
| *Phymaturus patagonicus* | Herbivorous | [1, 2, 11, 46, 47] |
| *Phymaturus payuniae* | Herbivorous | [1, 2, 7, 48] |
| *Phymaturus punae* | Herbivorous | [1, 2, 11, 36, 37, 49, 50] |
| *Phymaturus roigorum* | Herbivorous | [1, 48] |
| *Phymaturus sitesi* | Herbivorous | [1, 51] |
| *Phymaturus somuncurensis* | Herbivorous | [1, 2, 11] |
| *Phymaturus spurcus* | Herbivorous | [1, 2, 7] |
| *Phymaturus tenebrosus* | Herbivorous | [1, 3, 7, 36] |
| *Phymaturus verdugo* | Herbivorous | [1, 2, 7] |
| *Phymaturus videlai* | Herbivorous | [1, 52] |
| *Phymaturus vociferator* | Herbivorous | [1, 2, 25, 36, 53] |
| *Phymaturus williamsi* | Herbivorous | [1, 54] |
| *Phymaturus zapalensis* | Omnivorous | [1] |

* Personal data (i.e., records of stomach contents of different species) gathered by one of us (DPD).

+ Species that we reclassified from herbivorous to omnivorous

**References.**

1. Meiri S: **Traits of lizards of the world: Variation around a successful evolutionary design**. *Global Ecology and Biogeography* 2018, **27**(10):1168-1172.

2. Pincheira-Donoso D, Scolaro JA, Sura P: **A monographic catalogue on the systematics and phylogeny of the South American iguanian lizard family Liolaemidae (Squamata, Iguania)**. *Zootaxa* 2008, **1800**:1-85.

3. Cruz FB, Antenucci D, Luna F, Abdala CS, Vega LE: **Energetics in Liolaemini lizards: implications of a small body size and ecological conservatism**. *Journal of Comparative Physiology B* 2011, **181**:373-382.

4. Semham RV, Halloy M: **Diet and Reproductive States in a High Altitude Neotropical Lizard Species, Liolaemus ramirezae (Iguania: Liolaemidae)**. *Journal of Herpetology* 2016, **50**(4):589-593.

5. Schulte II JA, Losos JB, Cruz FB, Núñez H: **The relationship between morphology, escape behaviour and microhabitat occupation in the lizard clade *Liolaemus* (Iguanidae: Tropidurinae*: Liolaemini)**. *Journal Evolutionary Biology* 2004, **17**:408-420.

6. Pincheira-Donoso D, Núñez H: **Las especies chilenas del género *Liolaemus* Wiegmann, 1834 (Iguania, Tropiduridae: Liolaeminae) taxonomía, sistemática y evolución**. *Publicación Ocasional del Museo Nacional de Historia Natural* 2005, **59**:7-486.

7. Scolaro A: **Reptiles Patagonicos Norte. Una Guia de Campo**. Argentína: Universidad Nacional de la Patagonia San Juan Bosco; 2006.

8. Troncoso-Palacios J, Silva CR, Daniel TA: **Nuevos registros para dos especies de lagartos (*Liolaemus*) en la zona Central de Chile**. *La Chiricoca* 2011, **13**:9-13.

9. Demangel D: **Guia de Campo. Reptiles del centro sur de Chile**: Corporacion Chilena de la Madera (CORMA); 2016.

10. Semhan RV, Halloy M, Abdala CS: **Diet and reproductive states in a high altitude neotropical lizard, *Liolaemus crepuscularis* (Iguania: Liolaemidae)**. *South American Journal of Herpetology* 2013, **8**(2):102-108.

11. Espinoza RE, Wiens JJ, Tracy CR: **Recurrent evolution of herbivory in small, cold-climate lizards: Breaking the ecophysiological rules of reptilian herbivory**. *Proceeding of the National Academy of Sciences* 2004, **101**(48):16819-16824.

12. Scolaro A: **Reptiles patagónicos sur. Guía de campo**. Argentina: Universidad Nacional de la Patagonia San Juan Bosco; 2005.

13. Halloy S, Laurent RF: **Notes éco-éthologiques sur *Liolaemus huacahuasicus* Laurent (Iguanidae) du Nord-Ouest argentin**. *Revue Française d'Aquariologie* 1987, **14**:137-144.

14. Troncoso-Palacios J, Diaz HA, Puas GI, Riveros-Riffo E, Elorza AA: **Two new Liolaemus lizards from the Andean highlands of Southern Chile (Squamata, Iguania, Liolaemidae)**. *ZooKeys* 2016, **632**:121-146.

15. Scolaro JA, Tapari FO, Gonzáles C: **Phymaturus calcogaster: rectificación de la localidad tipo y descripción de la hembra (Reptilia, Iguania, Liolaemidae)**. *Facena* 2005, **21**:29-36.

16. Bonino MF, Moreno ADL, Tulli MJ, Abdala CS, Perotti MG, Cruz FB: **Running in cold weather: morphology, thermal biology, and performance in the southernmost lizard clade in the world (*Liolaemus lineomaculatus* Section: Liolaemini: Iguania)**. *Journal of Experimental Biology* 2011, **315**:495-503.

17. Rocha CFD: **Diet of a tropical lizard (Liolaemus lutzae) of Southeastern Brazil**. *Journal of Herpetology* 1989, **23**(3):292-274.

18. Rocha CFD: **Ontogenetic shift in the rate of plant consumption in a tropical lizard (Liolaemus lutzae)**. *Journal of Herpetology* 1998, **32**(2):274-279.

19. Rocha CFD: **Selectivity in plant food consumption in the lizard Liolaemus lutzae from southeastern Brazil**. *Stud Neotrop Fauna & Environm* 2000, **35**:14-18.

20. Cooper Jr. WE, Vitt LJ: **Distribution, extent, and evolution of plant consumption by lizards**. *Journal of Zoology* 2002, **257**:487-517.

21. Perry G, Garland Jr. T: **Lizard home ranges revisited: effects of sex, body size, diet, habitat, and phylogeny**. *Ecology* 2002, **83**(7):1870-1885.

22. Verrastro L, Veronese L, Bujes CS, Dias FMM: **A new species of *Liolaemus* from southern Brazil (Iguania: Tropiduridae)**. *Herpetologica* 2003, **59**(1):105-118.

23. Halloy M, Robles C, Cuezzo F: **Diet in two syntopic neotropical lizard species of *Liolaemus* (Liolaemidae): interspecific and intersexual differences**. *Revista Española de Herpetología* 2006, **20**:47-56.

24. Valladares JP, Etheridge R, Schulte II J, Manriquez G, Spotorno A: **Nueva especie de lagartija del norte de Chile, Liolaemus molinai (Reptilia: Liolaeminae)**. *Revista Chilena de Historia Natural* 2002, **75**:473-489.

25. Demangel D: **Reptiles en Chile**. Chile: Fauna Nativa Ediciones; 2016.

26. Jaksic FM, Fuentes ER: **Observaciones autoecológicas en *Liolaemus nitidus* (Lacertilia: Iguanidae)**. *Studies on Neotropical Fauna and Environment* 1980, **15**:109-124.

27. Stellatelli OA, Block C, Moreno-Azócar DL, Vega LE, Isacch JP, Cruz FB: **Scale dependency of Liolaemus lizards’ home range in response to different environmental variables**. *Current Zoology* 2016, **62**(6):521-530.

28. Verrastro L, Ely I: **Diet of the lizard *Liolaemus occipitalis* in the coastal sand dunes of southern Brazil (Squamata-Liolaemidae)** *Brazilian Journal of Biology* 2015, **75**(2):289-299.

29. Castillo GN, Acosta JC, Blanco GM: **Trophic analysis and parasitological aspects of *Liolaemus parvus* (Iguania: Liolaemidae) in the Central Andes of Argentina** *Turkish Journal of Zoology* 2019, **43**:277-286.

30. Valdecantos MS, Arias F, Espinoza RE: **Herbivory in *Liolaemus poecilochromus*, a Small, Cold-Climate Lizard from the Andes of Argentina**. *Copeia* 2012, **2**:203-210.

31. Lobo F, Espinoza RE: **Two new liolaemus from the puna region of Argentina and Chile: Further resolution of purported reproductive bimodality in *Liolaemus alticolor* (Iguania: Liolaemidae)**. *Copeia* 2004, **2004**(4):850-867.

32. Pearson OP, Bradford DF: **Thermoregulation of lizards and toads at high altitudes in Peru**. *Copeia* 1976, **1976**(1):155-170.

33. Pincheira-Donoso D, Scolaro JA: **Iguanian species-richness in the Andes of boreal Patagonia: Evidence for an additional new *Liolaemus* lizard from Argentina lacking precloacal glands (Iguania, Liolaeminae)**. *Zootaxa* 2007, **1452**:55-68.

34. Troncoso-Palacios J, Díaz HA, Esquerre D, Urra FA: **Two new species of the *Liolaemus elongatus-kriegi* complex (Iguania, Liolaemidae) from Andean highlands of southern Chile**. *ZooKeys* 2015, **500**:83-109.

35. Troncoso-Palacios J, Esquerré D: **A new species of Phymaturus of the *P. mallimaccii* group from tha Andes of central Chile**. *Phyllomedusa* 2014, **13**(1):3-15.

36. Boretto JM, Ibargüengoytía NR: **Phymaturus of Patagonia, Argentina: Reproductive Biology of *Phymaturus zapalensis* (Liolaemidae) and a Comparison of Sexual Dimorphism within the Genus**. *Journal of Herpetology* 2009, **43**(1):96-104.

37. Boretto JM, Cabezas-Cartes F, Ibargüengoytía NR: **Slow life histories in lizards living in the highlands of the Andes Mountains**. *Journal of Comparative Physiology B* 2017, **188**(3):491-503.

38. Troncoso-Palacios J, Lobo F, Etheridge R, Acosta JC, Laspiur A: **The rediscovery of *Oplurus bibronii* Guichenot, 1848 a valid species of the liolaemid genus *Phymaturus***. *Zootaxa* 2013, **3652**(5):547-561.

39. Lobo F, Nenda SJ, Slodki D: **A new lizard of Phymaturus (Iguania: Liolaemidae) from Argentina**. *Herpetologica* 2012, **68**(1):12-133.

40. Lobo F, Espinoza RE, Sanabria EA, Quiroga LB: **A New *Phymaturus* (Iguania: Liolaemidae) from the Southern Extreme of the Argentine Puna**. *Copeia* 2012, **2012**(1):12-22.

41. Scolaro A, Ibargüengoytía NR: **A new fragment for the understanding of the puzzling evolutive process of the *Phymaturus* genus: a new species of the *patagonicus* group from Patagonia, Argentina (Reptilia: Iguania: Liolaemidae)**. *Zootaxa* 2008, **1939**:38-50.

42. Cabezas-Cartes F, Boretto J, Acosta JC, Jahn G, Blanco G, Laspiur A, Ibargüengoytía N: **Reproductive biology of *Phymaturus* cf. *palluma*: a vulnerable lizard from the highlands of the Andes, San Juan, Argentina**. *Herpetological Conservation and Biology* 2010, **5**(3):430-440.

43. Eisenberg T, Werning H: ***Phymaturus* cf. *palluma* in captivity: observations on its reproduction and biology**. *Salamandra* 2012, **48**(4):1-9.

44. Castro SA, Laspiur A, Acosta JC: **Variación anual e intrapoblacional de la dieta de *Phymaturus* cf. *palluma* (Iguania: Liolaemidae) de los Andes centrales en Argentina**. *Revista Mexicana de Biodiversidad* 2013, **84**:1258-1265.

45. Vicenzi N, Massarelli R: **Phymaturus palluma (high mountain lizard). Diet.** *Herpetological Review* 2018, **49**(1):121.

46. Ibargüengoytía NR: **Field, selected body temperature and thermal tolerance of the syntopic lizards *Phymaturus patagonicus* and *Liolaemus elongatus* (Iguania: Liolaemidae)**. *Journal of Arid Environments* 2005, **62**:435-448.

47. Piantoni C, Ibargüengoytía NR, Cussac VE: **Age and growth of the Patagonian lizard Phymaturus patagonicus**. *Amphibia Reptilia* 2006, **27**:385-392.

48. Corbalán V, Debandi G: **Resource segregation in two herbivorous species of mountain lizards from Argentina** *Herpetological Journal* 2014, **24**:201-208.

49. Boretto JM, Cabezas-Cartes F, Ibargüengoytía NR: **Energy allocation to growth and reproduction in a viviparous lizard endemic to the highlands of the Andes, Argentina** *Journal of Zoology* 2015, **297**:77-86.

50. Córdoba MA, Acosta JC, Villavicencio HJ: **Análisis trófico de *Phymaturus punae* (Iguania: Liolaemidae): variaciónestacional y sexual en la región más austral de la puna Argentina**. *Revista Mexicana de Biodiversidad* 2015, **86**:1004-1013.

51. Avila LJ, Fulvio PCH: **Two new mountain lizard species of the *Phymaturus* genus (Squamata: Iguania) from northwestern Patagonia, Argentina**. *Zootaxa* 2011, **2924**:1-21.

52. Scolaro JA, Pincheira-Donoso D: **Lizards at the end of the world: Two new species of *Phymaturus* of the *patagonicus* clade (Squamata, Liolaemidae) revealed in southern Patagonia of Argentina**. *Zootaxa* 2010, **2393**:17-32.

53. Pincheira-Donoso D: **Una nueva especie del género *Phymaturus* (Iguania: Tropiduridae: Liolaemini) del centro-sur de Chile**. *Multequina* 2004, **13**:57-70.

54. Lobo F, Laspiur A, Acosta JC: **Description of new andean species of the genus *Phymaturus* (Iguania: Liolaemidae) from Northwestern Argentina**. *Zootaxa* 2013, **3683**(2):117-132.

**Pruned tree that was used in this study**

#NEXUS

[R-package APE, Tue Apr 26 15:58:01 2022]

BEGIN TAXA;

DIMENSIONS NTAX = 185;

TAXLABELS

Ctenoblepharys_adspersa

Liolaemus_abaucan

Liolaemus_albiceps

Liolaemus_alticolor

Liolaemus_andinus

Liolaemus_annectens

Liolaemus_anomalus

Liolaemus_araucaniensis

Liolaemus_archeforus

Liolaemus_atacamensis

Liolaemus_audituvelatus

Liolaemus_austromendocinus

Liolaemus_azarai

Liolaemus_baguali

Liolaemus_bellii

Liolaemus_bibronii

Liolaemus_bitaeniatus

Liolaemus_boulengeri

Liolaemus_buergeri

Liolaemus_burmeisteri

Liolaemus_calchaqui

Liolaemus_canqueli

Liolaemus_capillitas

Liolaemus_chacabucoense

Liolaemus_chacoensis

Liolaemus_chaltin

Liolaemus_chehuachekenk

Liolaemus_chiliensis

Liolaemus_chillanensis

Liolaemus_coeruleus

Liolaemus_confusus

Liolaemus_constanzae

Liolaemus_crandalli

Liolaemus_crepuscularis

Liolaemus_cristiani

Liolaemus_curicensis

Liolaemus_curis

Liolaemus_cuyanus

Liolaemus_cuyumhue

Liolaemus_cyanogaster

Liolaemus_darwinii

Liolaemus_dicktracyi

Liolaemus_donosobarrosi

Liolaemus_dorbignyi

Liolaemus_eleodori

Liolaemus_elongatus

Liolaemus_escarchadosi

Liolaemus_espinozai

Liolaemus_etheridgei

Liolaemus_fabiani

Liolaemus_famatinae

Liolaemus_fitzgeraldi

Liolaemus_fitzingerii

Liolaemus_fuscus

Liolaemus_gallardoi

Liolaemus_goetschi

Liolaemus_gracilis

Liolaemus_gravenhorstii

Liolaemus_grosseorum

Liolaemus_gununakuna

Liolaemus_hatcheri

Liolaemus_heliodermis

Liolaemus_hellmichi

Liolaemus_hermannunezi

Liolaemus_huacahuasicus

Liolaemus_inacayali

Liolaemus_insolitus

Liolaemus_irregularis

Liolaemus_isabelae

Liolaemus_janequeoae

Liolaemus_kingii

Liolaemus_kolengh

Liolaemus_koslowskyi

Liolaemus_kriegi

Liolaemus_laurenti

Liolaemus_lavillai

Liolaemus_leftrarui

Liolaemus_lemniscatus

Liolaemus_leopardinus

Liolaemus_lineomaculatus

Liolaemus_loboi

Liolaemus_lorenzmuelleri

Liolaemus_lutzae

Liolaemus_magellanicus

Liolaemus_maldonadae

Liolaemus_mapuche

Liolaemus_martorii

Liolaemus_melaniceps

Liolaemus_melanops

Liolaemus_molinai

Liolaemus_monticola

Liolaemus_morenoi

Liolaemus_multicolor

Liolaemus_multimaculatus

Liolaemus_neuquensis

Liolaemus_nigrocoeruleus

Liolaemus_nigromaculatus

Liolaemus_nigroviridis

Liolaemus_nitidus

Liolaemus_occipitalis

Liolaemus_olongasta

Liolaemus_orientalis

Liolaemus_ornatus

Liolaemus_pagaburoi

Liolaemus_parvus

Liolaemus_paulinae

Liolaemus_petrophilus

Liolaemus_pictus

Liolaemus_platei

Liolaemus_poconchilensis

Liolaemus_poecilochromus

Liolaemus_pseudoanomalus

Liolaemus_pseudolemniscatus

Liolaemus_puna

Liolaemus_punmahuida

Liolaemus_quilmes

Liolaemus_ramirezae

Liolaemus_riojanus

Liolaemus_robertmertensi

Liolaemus_rothi

Liolaemus_ruibali

Liolaemus_sagei

Liolaemus_salinicola

Liolaemus_sarmientoi

Liolaemus_saxatilis

Liolaemus_scapularis

Liolaemus_schroederi

Liolaemus_scorialis

Liolaemus_senguer

Liolaemus_septentrionalis

Liolaemus_signifer

Liolaemus_silvai

Liolaemus_silvanae

Liolaemus_sitesi

Liolaemus_somuncurae

Liolaemus_stolzmanni

Liolaemus_tari

Liolaemus_tehuelche

Liolaemus_telsen

Liolaemus_tenuis

Liolaemus_torresi

Liolaemus_tregenzai

Liolaemus_tristis

Liolaemus_umbrifer

Liolaemus_uniformis

Liolaemus_uptoni

Liolaemus_uspallatensis

Liolaemus_valdesianus

Liolaemus_vallecurensis

Liolaemus_velosoi

Liolaemus_villaricensis

Liolaemus_walkeri

Liolaemus_wiegmannii

Liolaemus_xanthoviridis

Liolaemus_yanalcu

Liolaemus_zabalai

Liolaemus_zapallarensis

Liolaemus_zullyae

Phymaturus_aguedae

Phymaturus_antofagastensis

Phymaturus_bibronii

Phymaturus_calcogaster

Phymaturus_ceii

Phymaturus_delheyi

Phymaturus_denotatus

Phymaturus_dorsimaculatus

Phymaturus_extrilidus

Phymaturus_indistinctus

Phymaturus_mallimaccii

Phymaturus_manuelae

Phymaturus_nevadoi

Phymaturus_palluma

Phymaturus_patagonicus

Phymaturus_payuniae

Phymaturus_punae

Phymaturus_roigorum

Phymaturus_sitesi

Phymaturus_somuncurensis

Phymaturus_spurcus

Phymaturus_tenebrosus

Phymaturus_verdugo

Phymaturus_videlai

Phymaturus_vociferator

Phymaturus_williamsi

Phymaturus_zapalensis

;

END;

BEGIN TREES;

TRANSLATE

1 Ctenoblepharys_adspersa,

2 Liolaemus_abaucan,

3 Liolaemus_albiceps,

4 Liolaemus_alticolor,

5 Liolaemus_andinus,

6 Liolaemus_annectens,

7 Liolaemus_anomalus,

8 Liolaemus_araucaniensis,

9 Liolaemus_archeforus,

10 Liolaemus_atacamensis,

11 Liolaemus_audituvelatus,

12 Liolaemus_austromendocinus,

13 Liolaemus_azarai,

14 Liolaemus_baguali,

15 Liolaemus_bellii,

16 Liolaemus_bibronii,

17 Liolaemus_bitaeniatus,

18 Liolaemus_boulengeri,

19 Liolaemus_buergeri,

20 Liolaemus_burmeisteri,

21 Liolaemus_calchaqui,

22 Liolaemus_canqueli,

23 Liolaemus_capillitas,

24 Liolaemus_chacabucoense,

25 Liolaemus_chacoensis,

26 Liolaemus_chaltin,

27 Liolaemus_chehuachekenk,

28 Liolaemus_chiliensis,

29 Liolaemus_chillanensis,

30 Liolaemus_coeruleus,

31 Liolaemus_confusus,

32 Liolaemus_constanzae,

33 Liolaemus_crandalli,

34 Liolaemus_crepuscularis,

35 Liolaemus_cristiani,

36 Liolaemus_curicensis,

37 Liolaemus_curis,

38 Liolaemus_cuyanus,

39 Liolaemus_cuyumhue,

40 Liolaemus_cyanogaster,

41 Liolaemus_darwinii,

42 Liolaemus_dicktracyi,

43 Liolaemus_donosobarrosi,

44 Liolaemus_dorbignyi,

45 Liolaemus_eleodori,

46 Liolaemus_elongatus,

47 Liolaemus_escarchadosi,

48 Liolaemus_espinozai,

49 Liolaemus_etheridgei,

50 Liolaemus_fabiani,

51 Liolaemus_famatinae,

52 Liolaemus_fitzgeraldi,

53 Liolaemus_fitzingerii,

54 Liolaemus_fuscus,

55 Liolaemus_gallardoi,

56 Liolaemus_goetschi,

57 Liolaemus_gracilis,

58 Liolaemus_gravenhorstii,

59 Liolaemus_grosseorum,

60 Liolaemus_gununakuna,

61 Liolaemus_hatcheri,

62 Liolaemus_heliodermis,

63 Liolaemus_hellmichi,

64 Liolaemus_hermannunezi,

65 Liolaemus_huacahuasicus,

66 Liolaemus_inacayali,

67 Liolaemus_insolitus,

68 Liolaemus_irregularis,

69 Liolaemus_isabelae,

70 Liolaemus_janequeoae,

71 Liolaemus_kingii,

72 Liolaemus_kolengh,

73 Liolaemus_koslowskyi,

74 Liolaemus_kriegi,

75 Liolaemus_laurenti,

76 Liolaemus_lavillai,

77 Liolaemus_leftrarui,

78 Liolaemus_lemniscatus,

79 Liolaemus_leopardinus,

80 Liolaemus_lineomaculatus,

81 Liolaemus_loboi,

82 Liolaemus_lorenzmuelleri,

83 Liolaemus_lutzae,

84 Liolaemus_magellanicus,

85 Liolaemus_maldonadae,

86 Liolaemus_mapuche,

87 Liolaemus_martorii,

88 Liolaemus_melaniceps,

89 Liolaemus_melanops,

90 Liolaemus_molinai,

91 Liolaemus_monticola,

92 Liolaemus_morenoi,

93 Liolaemus_multicolor,

94 Liolaemus_multimaculatus,

95 Liolaemus_neuquensis,

96 Liolaemus_nigrocoeruleus,

97 Liolaemus_nigromaculatus,

98 Liolaemus_nigroviridis,

99 Liolaemus_nitidus,

100 Liolaemus_occipitalis,

101 Liolaemus_olongasta,

102 Liolaemus_orientalis,

103 Liolaemus_ornatus,

104 Liolaemus_pagaburoi,

105 Liolaemus_parvus,

106 Liolaemus_paulinae,

107 Liolaemus_petrophilus,

108 Liolaemus_pictus,

109 Liolaemus_platei,

110 Liolaemus_poconchilensis,

111 Liolaemus_poecilochromus,

112 Liolaemus_pseudoanomalus,

113 Liolaemus_pseudolemniscatus,

114 Liolaemus_puna,

115 Liolaemus_punmahuida,

116 Liolaemus_quilmes,

117 Liolaemus_ramirezae,

118 Liolaemus_riojanus,

119 Liolaemus_robertmertensi,

120 Liolaemus_rothi,

121 Liolaemus_ruibali,

122 Liolaemus_sagei,

123 Liolaemus_salinicola,

124 Liolaemus_sarmientoi,

125 Liolaemus_saxatilis,

126 Liolaemus_scapularis,

127 Liolaemus_schroederi,

128 Liolaemus_scorialis,

129 Liolaemus_senguer,

130 Liolaemus_septentrionalis,

131 Liolaemus_signifer,

132 Liolaemus_silvai,

133 Liolaemus_silvanae,

134 Liolaemus_sitesi,

135 Liolaemus_somuncurae,

136 Liolaemus_stolzmanni,

137 Liolaemus_tari,

138 Liolaemus_tehuelche,

139 Liolaemus_telsen,

140 Liolaemus_tenuis,

141 Liolaemus_torresi,

142 Liolaemus_tregenzai,

143 Liolaemus_tristis,

144 Liolaemus_umbrifer,

145 Liolaemus_uniformis,

146 Liolaemus_uptoni,

147 Liolaemus_uspallatensis,

148 Liolaemus_valdesianus,

149 Liolaemus_vallecurensis,

150 Liolaemus_velosoi,

151 Liolaemus_villaricensis,

152 Liolaemus_walkeri,

153 Liolaemus_wiegmannii,

154 Liolaemus_xanthoviridis,

155 Liolaemus_yanalcu,

156 Liolaemus_zabalai,

157 Liolaemus_zapallarensis,

158 Liolaemus_zullyae,

159 Phymaturus_aguedae,

160 Phymaturus_antofagastensis,

161 Phymaturus_bibronii,

162 Phymaturus_calcogaster,

163 Phymaturus_ceii,

164 Phymaturus_delheyi,

165 Phymaturus_denotatus,

166 Phymaturus_dorsimaculatus,

167 Phymaturus_extrilidus,

168 Phymaturus_indistinctus,

169 Phymaturus_mallimaccii,

170 Phymaturus_manuelae,

171 Phymaturus_nevadoi,

172 Phymaturus_palluma,

173 Phymaturus_patagonicus,

174 Phymaturus_payuniae,

175 Phymaturus_punae,

176 Phymaturus_roigorum,

177 Phymaturus_sitesi,

178 Phymaturus_somuncurensis,

179 Phymaturus_spurcus,

180 Phymaturus_tenebrosus,

181 Phymaturus_verdugo,

182 Phymaturus_videlai,

183 Phymaturus_vociferator,

184 Phymaturus_williamsi,

185 Phymaturus_zapalensis

;

TREE * UNTITLED = [&R] (1:43.71502474,(((((((((((2:6.855025207,73:6.855025207):0.5671543438,((((((3:0.6742405857,68:0.6742405857):2.335099437,103:3.009340022):0.7586754132,(21:3.252300972,34:3.252300972):0.5157144635):0.5193450808,76:4.287360516):1.849283917,(48:1.035714827,116:1.035714827):5.100929606):0.3585677284,147:6.495212161):0.9269673891):1.078556033,(25:6.28634669,(((41:1.297438256,75:1.297438256):1.948255046,59:3.245693302):1.474716212,101:4.720409514):1.565937176):2.214388893):3.702343307,(((((18:2.446078946,129:2.446078946):1.683998026,((66:2.204190687,139:2.204190687):0.7713821468,(122:1.596679834,138:1.596679834):1.378892999):1.154504138):1.544071022,81:5.674147994):1.075808462,120:6.749956456):2.357730415,(64:3.776683116,134:3.776683116):5.331003755):3.095392019):0.6252976131,((((22:1.604011847,(89:1.230875101,92:1.230875101):0.3731367459):0.3999590444,(56:1.072842041,87:1.072842041):0.9311288501):1.679609719,(27:2.189865944,(53:0.9278233313,154:0.9278233313):1.262042613):1.493714666):3.089363778,(38:5.438215958,(43:2.359041349,86:2.359041349):3.07917461):1.334728431):6.055432115):0.7420424464,((7:1.467318358,112:1.467318358):10.04024865,((((13:3.490740333,153:3.490740333):3.051496916,126:6.542237249):1.040318248,((39:2.111212054,(94:0.5410256071,118:0.5410256071):1.570186447):4.514426959,123:6.625639013):0.956916484):2.179505377,(83:5.834718567,100:5.834718567):3.927342308):1.745506136):2.062851939):1.440035511,(((((((5:2.009838972,111:2.009838972):2.55478346,93:4.564622431):1.982805888,44:6.547428319):0.3924287779,((67:2.158755081,136:2.158755081):3.717166284,(90:4.633993872,(110:1.759888334,141:1.759888334):2.874105538):1.241927493):1.063935733):0.5970622274,(((6:1.358782623,49:1.358782623):2.692115276,131:4.050897899):1.330911492,65:5.381809391):2.155109933):1.263054766,((((11:2.144281622,50:2.144281622):0.2601752866,(45:0.5134882806,149:0.5134882806):1.890968628):2.373509099,121:4.777966008):0.945565876,51:5.723531884):3.076442207):0.4938511586,102:9.29382525):5.716629211):4.622315264,(((((((((9:1.072061686,24:1.072061686):0.1096696549,143:1.181731341):0.330467256,158:1.512198597):0.820133127,(55:1.650484629,124:1.650484629):0.6818470946):0.7652477901,(14:1.357398624,(47:0.733942809,137:0.733942809):0.623455815):1.74018089):1.220462423,71:4.318041937):1.682087013,(135:3.894095877,146:3.894095877):2.106033074):2.48956215,84:8.4896911):2.911601682,((80:6.020821457,(72:1.469336677,133:1.469336677):4.551484781):2.452175519,61:8.472996977):2.928295806):8.231476942):5.481340812,(((((16:9.906228956,(((4:4.416129912,(26:2.910156064,114:2.910156064):1.505973848):2.133094637,(17:4.293428369,104:4.293428369):2.25579618):1.384080336,(((57:0.3118064206,125:0.3118064206):5.273788647,119:5.585595067):1.041943674,(117:1.703024795,155:1.703024795):4.924513946):1.305766144):1.972924071):2.554418876,(77:4.397372712,151:4.397372712):8.06327512):1.140015999,(((((8:2.311549366,108:2.311549366):1.073399604,36:3.38494897):3.329729047,(35:6.099775713,130:6.099775713):0.6149023044):1.011855422,(((15:2.223325652,52:2.223325652):2.107083528,((40:1.477013554,127:1.477013554):0.9707410062,58:2.447754561):1.88265462):2.470946561,28:6.801355741):0.9251776984):3.9861517,(30:0.6097337434,95:0.6097337434):11.1029514):1.887978691):4.177031138,(((((((29:2.104041885,128:2.104041885):0.5538571286,33:2.657899013):0.4719049219,(46:2.319718277,70:2.319718277):0.8100856579):0.7615515428,20:3.891355478):1.74356222,(((19:1.972937633,(74:1.83024534,156:1.83024534):0.1426922925):0.4900607484,142:2.462998381):1.396012975,(37:3.537953354,(79:0.265548489,148:0.265548489):3.272404865):0.3210580025):1.775906341):4.00031162,((((12:3.767790342,105:3.767790342):2.227975135,60:5.995765477):1.770266532,(((23:0.4351946494,144:0.4351946494):1.201720956,62:1.636915605):2.94103818,42:4.577953785):3.188078224):0.5226786238,107:8.288710632):1.346518685):3.436238885,115:13.0714682):4.706226767):3.809462966,((((((10:3.177193139,97:3.177193139):1.382821919,88:4.560015058):1.699289399,(132:2.563170729,157:2.563170729):3.696133728):8.523209945,((((32:1.000012245,69:1.000012245):2.730787404,106:3.730799649):3.675502515,(((63:4.159840056,((96:0.564994625,150:0.564994625):2.476947685,109:3.04194231):1.117897747):2.006366143,82:6.166206199):0.412163654,85:6.578369853):0.827932311):5.734199466,113:13.14050163):1.642012771):2.821033395,(((31:7.096311664,(91:4.646919852,99:4.646919852):2.449391813):4.933316969,(54:9.388931211,(98:4.620412495,145:4.620412495):4.768518716):2.640697422):2.818653718,78:14.84828235):2.755265445):1.048069416,140:18.65161721):2.935540721):3.526952602):0.9697475235,152:26.08385806):13.76766472,((((159:2.012520626,((((160:0.451527901,165:0.451527901):0.2480968525,169:0.6996247534):0.3373128098,175:1.036937563):0.1436384305,((161:0.5549294058,184:0.5549294058):0.1721829306,167:0.7271123363):0.4534636573):0.831944632):0.5028702771,((181:0.1888287469,172:0.1888287469):0.4032365488,176:0.5920652957):1.923325607):2.080581143,(166:0.4382694681,183:0.4382694681):4.157702577):12.21129256,((((162:1.666393113,(163:0.5957680731,178:0.5957680731):1.07062504):0.5089269864,180:2.1753201):2.375642005,(((164:0.905686699,((171:0.3421251657,174:0.3421251657):0.1323552341,177:0.4744803997):0.4312062992):2.052279282,185:2.957965981):0.3412618061,((179:1.010409573,170:1.010409573):0.6586393712,173:1.669048944):1.630178843):1.251734318):0.5554505247,(168:0.2738087942,182:0.2738087942):4.832603835):11.70085197):23.04425818):3.863501958);

END;
